# Validation of the Novel Web-Based Application HUMTELEMED for a Comprehensive Assessment of Cardiovascular Risk Based on the 2021 European Society of Cardiology Guidelines

**DOI:** 10.3390/jcm13082295

**Published:** 2024-04-16

**Authors:** Matteo Landolfo, Francesco Spannella, Alessandro Gezzi, Federico Giulietti, Lucia Sabbatini, Isabella Bari, Romina Alessandroni, Angelica Di Agostini, Paolo Turri, Francesco Alborino, Lorenzo Scoppolini Massini, Riccardo Sarzani

**Affiliations:** 1 Internal Medicine and Geriatrics, Istituti di Ricovero e Cura a Carattere Scientifico (IRCCS), Istituto Nazionale di Ricovero e Cura per gli Anziani (INRCA), 60127 Ancona, Italy; m.landolfo@inrca.it (M.L.); a.gezzi@inrca.it (A.G.); f.giulietti@inrca.it (F.G.); l.sabbatini@inrca.it (L.S.); i.bari@inrca.it (I.B.); r.alessandroni@inrca.it (R.A.); a.diagostini@inrca.it (A.D.A.); p.turri@inrca.it (P.T.); r.sarzani@univpm.it (R.S.); 2 Department of Clinical and Molecular Sciences (DISCLIMO), Centre of Obesity, University “Politecnica delle Marche”, 60127 Ancona, Italy; 3 AIDAPT Srl, 60127 Ancona, Italy; francesco.alborino@aidaptsrl.com (F.A.); lorenzo.scoppolinimassini@aidaptsrl.com (L.S.M.)

**Keywords:** cardiovascular risk, SCORE2, SCORE2-OP, humtelemed, hypertension, dyslipidemia

## Abstract

**Background and aims:** SCORE2/SCORE2-OP cardiovascular risk (CVR) charts and online calculators do not apply to patients with comorbidities, target organ damage, or atherosclerotic cardiovascular disease, for whom the assessment relies on the conventional consultation of the 2021 ESC guidelines (qualitative approach). To simplify the CVR evaluation, we developed an integrated multi-language and free-to-use web application. This study assessed the agreement between the conventional method versus our web app. **Methods:** A cross-sectional study was carried out on 1306 consecutive patients aged 40+ years referred to our center for the diagnosis and management of hypertension and dyslipidemia. Two double-blind operators performed the CVR assessment and classified each patient into low–moderate-, high-, and very-high-risk categories by using the conventional method (SCORE2/SCORE2-OP charts and consultation of the 2021 ESC guidelines) and the web app. The Kappa statistics were used to compare the two methods. **Results**: The mean age was 60.3 ± 11.9 years, with male prevalence (51.4%). Patients in primary prevention were 77.0%. According to the SCORE2/SCORE2-OP charts and 2021 ESC guideline consultation, the CVR was low–moderate in 18.6% (n° 243), high in 36.8% (n° 480), and very high in 44.6% (n° 583). According to the web app, individual CVR was low–moderate in 19.5% (n° 255), high in 35.4% (n° 462), and very high in 45.1% (n° 589). The two methods strongly agreed (Kappa = 0.960, *p* < 0.001), with a 97.5% concordance. **Conclusions**: our application has excellent reliability in a broad “real life” population and may help non-expert users and busy clinicians to assess individual CVR appropriately, representing a free-to-use, simple, time-sparing and widely available alternative to the conventional CVR evaluation using SCORE2/SCORE2-OP and 2021 ESC guideline charts.

## 1. Introduction

Cardiovascular risk (CVR) assessment is pivotal in identifying patients deserving of prompt and effective preventive strategies and therapies. Based on the results of numerous epidemiological and clinical studies in recent decades, individual CVR assessment has become more precise, accounting for emerging cardiovascular disease (CVD) determinants, such as country of origin, sex, race, and others. These data have also been used to develop tools to assess individual CVR. The first CVR calculator with a correspondent web version was derived from the Framingham Heart Study and is still available online (www.framinghamheartstudy.org, accessed on 24 February 2024). In this regard, the latest 2021 European Society of Cardiology (ESC) guidelines on the prevention of cardiovascular disease endorsed the use of the Systematic COronary Risk Evaluation (SCORE) in its updated version (SCORE2) and its counterpart designed for people over 70 years (SCORE2-OP) [1,2]. Both SCORE2 and SCORE2-OP estimate 10-year fatal and non-fatal CVD risk in European cohorts of individuals over 40 years by taking into account the country of origin, sex, age, and smoking habit, combined with non-high-density lipoprotein cholesterol (non-HDL-C) and office systolic blood pressure (SBP), as measurements of total atherogenic circulating cholesterol and arterial BP burden, respectively [3]. Individual CVR through SCORE2/SCORE2-OP can be estimated by physicians in daily clinical practice using web applications, such as HeartScore (www.heartscore.org, accessed on 24 February 2024) or U-prevent (https://u-prevent.com, accessed on 24 February 2024), available only in English and Dutch. These online calculators also provide several “secondary” CVR scores for specific subpopulations (i.e., patients with previous CVD, type 2 diabetes mellitus), in addition to SCORE2/SCORE2-OP, the only one considered by 2021 ESC guidelines for the assessment of global CVR classification with clinical and therapeutic impact. However, the results of SCORE2/SCORE2-OP are applicable only in primary prevention and in patients without significant CV comorbidities or related target organ damage (TOD). Indeed, the presence of chronic kidney disease (CKD), familial hypercholesterolemia (FH), diabetes mellitus (DM), or documented clinical and instrumental data regarding atherosclerotic cardiovascular disease (ASCVD) makes SCORE2/SCORE2-OP not applicable since these conditions already identify specific patient CVR categories (low–moderate, high, and very high through a qualitative approach), as pointed out by the ESC 2021 guidelines [3]. This complexity may lead to possible inertia and error made by less expert physicians and a consequent underestimation of CVR in some populations. To simplify individual CVR assessment in daily clinical practice, especially for non-expert users and busy or non-English-friendly physicians, we developed a free-to-use web application (www.humtelemed.it) based on the 2021 ESC guideline CVR classification taking into account both SCORE2/SCORE2-OP and patient’s CV comorbidities. This web app is the first that integrates this entire clinical decision process (SCORE2/SCORE2-OP charts and qualitative approach), as well as allowing for the automatic calculation of the estimated glomerular filtration rate (eGFR) by CKD-EPI equation, low-density lipoprotein cholesterol (LDL-C), and non-HDL-C, into a single procedure for the user. This study assessed the agreement in individual CVR classification between the one estimated through the “manual” and time-consuming conventional method (SCORE2/SCORE2-OP charts and the 2021 ESC guideline consultation) versus the one estimated by the multi-language web app.

## 2. Materials and Methods

### 2.1. Study Population and Design

We conducted a cross-sectional study on 1306 consecutive outpatients referred to our “Hypertension Excellence Centre” of the European Society of Hypertension (ESH) from January 2022 to September 2023 due to high BP and/or dyslipidemia first evaluation and management. Although it is an excellent specialistic center, most patients are referred by general practitioners and a minority by other specialists. Therefore, it reflects the general population of our region/country. All patients enrolled were adults aged ≥40 years in primary or secondary CV prevention. The lack of essential data for CV risk assessment, such as anthropometric measurements, accurate BP measurements, and routine lab examinations, was considered an exclusion criterion. All participants gave their informed consent, and clinical investigations were conducted according to the principles of the Declaration of Helsinki and its later amendments. This study was approved by the local institutional ethics committee (INRCA ethics committee). 

### 2.2. Individual CVR Stratification 

For the individual 10-year fatal and non-fatal CV event risk, the web app has been calibrated with SCORE2/SCORE2-OP for moderate-risk European regions, which include Italy, and by taking into account concomitant CVR factors or ASCVD, as suggested by the 2021 ESC guidelines on cardiovascular disease prevention in clinical practice (Table 1) [3]. Two double-blind, expert physicians (LS and AG), who work daily at our hypertension center, performed the CVR assessment and classified each patient into low–moderate-, high-, or very-high-risk categories. One operator used the conventional method of assessing SCORE2/SCORE2-OP with online calculators, risk charts, or the qualitative approach through visual consultation of the 2021 ESC guidelines (Table 1), whenever necessary (i.e., patients with DM or CKD), as usually carried out in daily clinical practice. The second operator instead evaluated the same patients using the web app www.humtelemed.it (available in Italian, English, and French). The web app is not a new calculator. Its risk algorithm, developed in collaboration with the engineers of the AIDAPT SRL (Ancona, Italy) as a spin-off of a start-up project of the Polytechnic University of Marche (Ancona, Italy), simultaneously provides individual CVR stratification by combining the following variables: age, sex, country of origin, body mass index (BMI, Kg/m^2^), actual or previous smoking habit, SBP, diastolic blood pressure (DBP), total cholesterol (TC), HDL-C, triglycerides (TGs), LDL-C (calculated using the Friedewald equation modified by Martin et al. [4]), creatinine, eGFR by CKD-EPI equation, ASCVD (clinical or imaging), familial hypercholesterolemia (FH), DM, presence of microalbuminuria, and current lipid-lowering therapy (LLT) or antihypertensive treatment. In particular, the algorithm works as follows: if the patient is in primary prevention without comorbidities directly affecting the CV risk stratification according to ESC guidelines [3], as also briefly summarized in Table 1, the CV risk is estimated by SCORE2/SCORE2-OP; on the other hand, if the patient suffers from CKD or DM, has a markedly elevated single risk factor, is affected by familial hypercholesterolemia, or has documented ASCVD (secondary prevention), the CV risk is estimated qualitatively (low–moderate, high, very high) according to 2021 ESC guidelines on cardiovascular disease prevention in clinical practice charts [3], summarized in Table 1. An example of the output of the web app is available in Supplemental Table S1. The parameters available or requested during the clinical evaluation were used to calculate the individual CVR. Of note, some data regarding the duration of DM and additional laboratory or imaging information of TOD (i.e., urine albumin-to–creatinine ratio, cardiac or vascular ultrasound evaluation, etc.) were unavailable for some patients for further refinement of CVR assessment, as often occurs in everyday clinical practice. This lack of such variables affected both CVR assessment methods and did not alter our findings. 

### 2.3. Development of the Web App “www.humtelemed.it“

Humtelemed’s platform is divided into backend and frontend components.

#### 2.3.1. Frontend

The frontend of Humtelemed is developed in React.js using TypeScript. Zustand was adopted for global state management, while MUI was used as the component library. As part of the frontend development of the Humtelemed platform, the adoption of React.js and TypeScript represents a methodological choice that aims to optimize component interoperability and type safety of the code. React.js facilitates the encapsulation of behaviors and interfaces in discrete components, promoting code reusability and modularity, which are fundamental elements for the maintenance and evolution of complex software systems. TypeScript, with its static type system, helps reduce runtime errors through type validation at compile time, significantly improving code quality and maintainability. Global state management, entrusted to Zustand, is distinguished by its effectiveness and simplicity, facilitating data exchange between non-hierarchically connected components without the overhead typical of other state management solutions. This approach aligns with modern frontend development practices, where efficient state management proves critical to ensure optimal performance and a smooth user experience. MUI (Material UI) to define UI components is part of a system-oriented design strategy, which pursues visual consistency and usability through a predefined set of design rules and standardized UI components. MUI facilitates the adoption of inclusive and accessible design principles, ensuring that the platform is usable by a broad spectrum of users, including those with disabilities.

#### 2.3.2. Backend

Humtelemed’s backend architecture, implemented with ASP.NET, forms the operational core of the platform, ensuring scalability, security, and flexibility. This technology choice enables the efficient integration of essential services such as token-based authentication, which is crucial for security, user session management, and sophisticated data management through MongoDB. Under its schemeless nature, the latter enables dynamic data management, critical in domains characterized by significant information variability such as health care. The backend handles critical tasks such as registering and authenticating users, sending e-mails for enrollment and password retrieval, and managing information about pharmacies and completions made by pharmacists. These operations are vital to the Humtelemed ecosystem, as they enable secure and private processing of health information and facilitate communication between different stakeholders. The generation of PDF documents (Report S1), which summarize the compilations made, is a crucial functionality, improving interoperability with existing systems and providing users with a direct means of sharing information. This data processing and presentation capability underscores the importance of a robust, well-designed backend that handles application logic and supports data presentation in accessible and functional formats.

### 2.4. Blood Pressure Measurement

During office evaluation, three sequential oscillometric automatic BP measurements, with a minute interval, were performed simultaneously on both arms using validated devices (Microlife^®^ model BP3MQ1-2D and BP A200 AFib, Widnau, Switzerland). Correct cuff sizes (range 22–32 cm, 32–42 cm, and 32–52 cm) were selected according to arm circumference, and BP measurements were performed after at least 5 min of rest in the sitting position. The patient’s arm, leaning on a desk or medical bed, was kept at the heart level during the measurements. The higher average SBP value between arms was used for the CVR assessment, thus avoiding errors due to interarm BP differences [5]. Hypertension was defined as the presence of antihypertensive therapy or SBP ≥ 140 mmHg and/or DBP ≥ 90 mmHg on at least two different occasions. 

### 2.5. Clinical and Laboratory Parameters

We collected all recruited patients’ medical histories, anthropometric measurements (BMI, defined as the body mass divided by the square of the body height and expressed in units of kilogram per square meter, and waist circumference), CV drug therapy, and complete laboratory tests. The CV drug therapy, including antihypertensive and lipid-lowering therapy (LLT) medications, was based on the self-report of patients and their confirmation during the clinical visit. We considered the main antihypertensive drug classes (RAAS inhibitors, calcium-channel blockers, diuretics, beta- and alpha-blockers, and mineralocorticoid receptor antagonists) as antihypertensive treatments. Statins, ezetimibe, and PCSK9 inhibitors and fibrates were considered for the LLT. The CKD-EPI creatinine equation estimated the glomerular filtration rate (eGFR). The lipid profile was obtained after fasting sampling, and then LDL-C was calculated using the Friedewald equation modified by Martin [4]. Non-HDL-C was calculated by subtracting the HDL-C from the TC. DM was defined based on documented medical history, the use of antidiabetic drugs confirmed during the clinical visit, or the detection of glycated haemoglobin ≥6.5% in routine lab examinations or repeated basal glycaemia ≥126 mg/dl. Smoking status was ascertained during recruitment, and the smoking habit was defined as the current or previous smoking of at least 100 cigarettes in a lifetime.

### 2.6. Statistical Analysis

Continuous variables were checked for normality and expressed as mean ± standard deviation (SD) or median and interquartile range (IQR) if markedly skewed. Categorical variables were expressed as numbers and percentages. The analysis of variance (ANOVA), Kruskal–Wallis test and χ^2^ test were used to analyze the differences in the main characteristics between patients with different CVRs. The marginal homogeneity test was used to evaluate the differences between the method’s results. The Kappa statistic was used to compare the agreement between the two methods. A *p*-value < 0.05 was considered statistically significant. All statistical analyses were conducted with SPSS version 23 [SPSS Inc., Chicago, IL, USA], Microsoft Windows version. 

## 3. Results

The general characteristics of the study population are reported in Table 2. We included 1306 patients in the analyses. The mean age was 60.3 ± 12.0 years, and male prevalence was 51.4%. The mean BMI was 28.3 ± 11.5 kg/m^2^, and the mean waist circumference was 95.0 ± 10.1 cm in females and 102.8 ± 11.1 cm in males, respectively. Obese (OB) patients (BMI ≥ 30 kg/ m^2^) were 32.2%. The prevalence of DM was 12.6%. The mean office SBP was 132.7 ± 15.3 mmHg, and the mean office DBP was 78.5 ± 10.6 mmHg. The prevalence of arterial hypertension was 65.5%. Patients in primary prevention were 1005 (77.0% of the study population). Regarding pharmacological treatment, 34.3% were on LLT and 45.4% on antihypertensive therapy. Regarding the serum lipid profile, the mean TC was 194.1 ± 43.2 mg/dL, mean HDL-C was 54.9 ± 15.2 mg/dL, and median for TGs was 104 (IQR 78–146) mg/dL, respectively. Mean LDL-C values were 114.8 ± 39.0 mg/dL. The mean eGFR (calculated by the CKD-EPI equation) was 78.5 ± 19.5 mL/min/1.73 m^2^, and the prevalence of CKD (eGFR < 60 mL/min/1.73 m^2^) was 8.0%. Individual CVR estimation using conventional SCORE2/SCORE2-OP charts and 2021 ESC guidelines consultation classified 243 patients (18.6%) into low–moderate risk, 480 patients (36.8%) into high risk, and 583 patients (44.6%) into very high risk. Table 3 shows the characteristics of the study population according to CVR stratification. As expected, the higher the risk, the higher the age, BP values, and the prevalence of the main comorbidities, such as DM, smoking, and CKD, and the lower the values of eGFR and HDL-C. Patients in the very high CVR group were more treated with LLT. 

### Agreement between Conventional CVR Assessment and www.humtelemed.it

Individual CVR estimation using the web app www.humtelemed.it found 255 patients (19.5%) at low–moderate risk, 462 patients (35.4%) at high risk, and 589 patients (45.1%) at very high risk. According to the marginal homogeneity test, the individual CVR assessed using the web app www.humtelemed.it was not statistically different from the one evaluated using the SCORE2/SCORE2-OP charts and the conventional 2021 ESC guideline consultation (*p* = 0.907). The two methods had a significant agreement (Kappa = 0.960, *p* < 0.001) since they agreed in 97.5% of cases (Table 3). The analyses revealed more disagreement within the middle (high-risk) CVR category. The 3.1% (n = 15) and the 2.1% (n = 10) of patients classified as high-risk by the conventional method fell into the low-moderate- and very-high-risk categories, respectively, when the CVR was assessed by www.humtelemed.it. 

To better investigate in which populations the disagreement occurred, the agreement between the conventional CVR assessment and www.humtelemed.it has also been tested in the following subgroups: age (above and below 65 years); sex (males and females); and presence of DM, CKD, and primary/secondary prevention. As reported in Table 4, all Kappa statistics in these subgroups maintained a significant agreement. In almost all subgroups, the degree of agreement was virtually perfect and comparable to that found in the general population (Kappa > 0.950), except for subjects with CKD or DM (Kappa = 0.736 and Kappa = 0.862, respectively), who showed a moderate/strong agreement [6]. In these two subgroups, the disagreement was only present in patients classified in the middle category (high CVR) by the conventional assessment, in which the web app tended to estimate a lower risk in the first case (patients with eGFR < 60 mL/min/1.73 m^2^) and a higher risk in the second group (patients with DM) (Supplemental Tables S2 and S3). 

## 4. Discussion

Our study aimed to test the reliability of the free-to-use web app www.humtelemed.it in a comprehensive “real life” population of patients at different CVR categories to prove its applicability in daily clinical practice. 

Overcoming the poor achievement of the guidelines’ recommended targets in patients at any stage of CVR is a worldwide, major clinical challenge that is still far from being addressed in primary prevention and ASCVD scenarios [7,8,9]. Yet, in recent years, several innovative drug treatments have become available to clinicians, especially with regard to dyslipidemia, such as bempedoic acid, human monoclonal antibodies against proprotein convertase subtilisin/kexin type 9 (PCSK9), and inclisiran, allowing for multiple combined LLTs to reach an LDL-C reduction of even more than 80% compared to that of the baseline [10,11]. Others are also emerging on the arterial hypertension front with the development of aldosterone synthase inhibition and zilebesiran, the first small interfering RNA (siRNA) designed to reduce angiotensinogen mRNA in the hepatocytes [12,13]. 

While patients’ lack of adherence remains a prominent factor, healthcare professionals’ responsibility has been more recently brought to light. Several important physician-related actionable factors require improvement to close the gap between guideline recommendations and real-world clinical practice [14,15]. There are multiple reasons behind this unsolved issue; among them, a major one is inaccurate initial CVR assessment [16]. Also, in real-life clinical practice in many countries, including Italy, the routine use of charts or similar tools to estimate CV risk is limited to a low percentage of practicing physicians [17]. Overall, in the SANTORINI study [18], which was an observational study conducted on patients with high and very high CVR in 14 European countries between 2020 and 2021, only 20.1% of patients achieved risk-based LDL-C goals as per the 2019 ESC/EAS guidelines, with a maximum of 31% of the patients receiving combination therapy (24% of the study population). In this study, the patients with the worst prevalence of the LDL-C goal were those with very high risk without ASCVD (12.3% of patients at goal), with those patients likely requiring careful CVR evaluation for their appropriate management. Surprisingly, only half (52.0%) of the physicians who participated in the study had used the ESC/EAS European guidelines for CVR classification. In comparison, 34.2% followed their clinical experience to evaluate whether a patient had high or very high CVR. Consequently, 15.7% of patients with documented ASCVD were misclassified as only at high risk. A stimulating survey has been conducted on 67 Italian outpatient services involving endocrinologists, internists, and geriatric practitioners dealing with CVR estimation and LDL-C management in patients with DM. The authors found that the assessment of CVR by physicians was comparable to that suggested by guidelines in two-thirds of cases. Thus, 35% of cases that were wrongly identified as CVR ones were exposed to harms related to suggested LDL-C targets higher than those recommended by guidelines and related to consequent less intensive lipid-lowering therapy (LLT). Subjects with a misclassified CVR assessment had a 67% lower probability of achieving the LDL-C goal than those with well-classified CVR (OR 0.33; 95% CI 0.23–0.46), independent of CVR categories. The most relevant factor associated with a better CVR classification was the presence of ASCVD, which reduced the probability of an inaccurate risk assessment by 88% (OR 0.12, 95% CI 0.06–0.21, *p* < 0.0001) and at the same time increased the likelihood of receiving high- or very-high-intensity LLT (OR 4.02, 95% CI 3.17–5.08, *p* < 0.0001), leading to 71% higher odds of achieving the LDL-C goal (OR 1.71, 95% CI 1.27–2.32, *p*  =  0.001) [19]. These latter data also suggested that the failure to classify patients’ CVR appropriately is more relevant in primary prevention, in which the web app www.humtelemed.it shows excellent reliability. The lack of a more comprehensive evaluation that includes all those additional risk factors, such as CKD and albuminuria, or other markers of target organ damage (TOD) could be partly responsible in this clinical setting. Thus, providing physicians, especially general practitioners, with simple and reliable tools to assess CVR more broadly in daily clinical practice may reverse these trends. In this regard, web-based calculators, such as the ESC-endorsed HeartScore (www.heartscore.org) and U-prevent (https://u-prevent.com), are widely available but not adequately implemented in clinical practice, despite the fact they may improve both patients’ self-management and physicians’ decision-making process concerning the initiation or implementation of therapeutical strategies, consequently impacting outcomes and cost-effectiveness [20]. To facilitate the consultation of these calculators, which do not allow for an integrated and global vision of the CVR assessment as our web app www.humtelemed.it does, the ESC released a specific ESC app for smartphones for easy and quick use in everyday clinical practice. 

In Europe, all web-based and validated tools to estimate CVR in primary prevention are limited to risk algorithms based on SCORE2/SCORE2-OP, only accounting for basal risk factors such as age, sex, smoking habit, and lipid and blood pressure profiles. In patients with comorbidities (i.e., obesity, CKD, DM), TOD, ASCVD, and multiple pharmacotherapies, scores and online calculators could be non-applicable or must be interpreted with care, as they can underestimate CVR [21]. The visual consultation of the 2021 ESC guidelines is time-wasting and often comes with struggles related to lack of experience and poor English understanding. 

Recently, while our data collection and investigation were ongoing, SCORE2-Diabetes [21] and SCORE2 with a CKD add-on [22] were developed and validated to improve CVR prediction in DM and CKD populations, respectively. Until now, in clinical practice, the global CVR in these two population subgroups was only assessed using a qualitative approach, as stated in Table 4 of the 2021 ESC guidelines [5]. Our web app’s main novelty lies in integrating the SCORE2/SCORE2-OP calculator with this qualitative approach for selected populations in a single application, which is easier to use in everyday clinical practice. In the meantime, we are developing continuous updates for the web app, and we will also consider these new specific calculators for DM and CKD in the future. 

By using the web app www.humtelemed.it, physicians or any user may easily overcome the conventional consultation and interpretation of the 2021 ESC guidelines, a significant limitation in daily clinical practice, obtaining reliable and almost perfectly matchable results, as emerged from our analyses regarding the agreement between the two methods. Disagreement was found in only 2.5% of cases. However, it is essential to point out that, in a vast population, a certain percentage of error can also be linked to the conventional CVR estimation, as previously described. Indeed, in a minority of cases in large populations, casual human errors in risk estimation cannot be completely ruled out. This aspect could at least partially justify the slight disagreement between the two methods, especially concerning individuals in the “medium” high-risk category affected by CKD or DM, in which unavailable or misinterpreted data about additional risk determinants play an essential role in classification (see Table 1). 

### Study Strengths and Limitations

The strengths of our study are the broad population taken into account and the setting where the experimentation took place: a “Hypertension Excellence Centre” of the ESH with daily and high-level experience in managing hypertension, dyslipidemia, and CVR evaluations. However, our study has some limitations. Despite the numerosity of the study sample, confirmation of these findings on larger scales is necessary. The web app is currently free to use and available to thousands of users who have already used it. Our study is designed to evaluate the degree of accuracy and reliability of our web app compared to that of clinical routine assessment. In this setting, there is no availability of a tool which can be considered as a proper gold standard. As a consequence, the non-infallibility of the conventional CVR evaluation due to human errors cannot be completely ruled out. This does not allow us to understand whether the small amount of CV misclassification found is due to the web app or to an error in the “manual” CVR evaluation method. 

## 5. Conclusions

Correctly estimating CVR in individual subjects is the first step in preventing CV events and death. Given the widespread prevalence of these health problems in the general population, reliable, time-sparing, and straightforward tools that are easily applicable even by non-expert operators can help in daily practice and should be disseminated on a large scale. Our multi-language, web-based app, which unifies all the data suggested by the 2021 ESC guidelines, was explicitly developed for this purpose. It shows almost perfect agreement with usual care and excellent reliability. The web app www.humtelemed.it could improve awareness of CVR in the general population and increase the diffusion on a large scale of a proper CV risk estimation, as recommended by the European guidelines on CV prevention. 

## Figures and Tables

**Table 1:** 2021 ESC guidelines cardiovascular risk stratification (10-year risk of fatal and non-fatal CV events).

Cardiovascular Risk	
**Low–Moderate**	- SCORE2 < 2.5% if age < 50 years - SCORE2 < 5% if age 50–69 years - SCORE2-OP < 7.5% if age ≥ 70 years - T1DM if age > 40 years - T2DM < 10 years of duration without other CV risk factors or TOD
**High**	- SCORE2 2.5–7.5% if age < 50 years - SCORE2 5–10% if age 50–69 years - SCORE2-OP 7.5–15% if age ≥ 70 years - Markedly elevated single risk factor (TC > 310 mg/dL or LDL-C > 190 mg/dL or BP ≥ 180/110 mmHg) - FH without other risk factors - eGFR 30–44 mL/min and ACR < 30 mg/g - eGFR 45–60 mL/min and ACR 30–300 mg/g - eGFR ≥ 60 mL/min and ACR > 300 mg/g - T2DM > 10 years of duration without TOD or with one other risk factor
**Very High**	- ASCVD (Clinical/Imaging) - SCORE2 ≥ 7.5% if age < 50 years - SCORE2 ≥ 10% if age 50–69 years - SCORE2-OP ≥ 15% if age ≥ 70 years - eGFR < 30 mL/min - eGFR 30–44 mL/min and ACR > 30 mg/g - T2DM with TOD

SCORE = Systematic COronary Risk Evaluation; T1DM = type 1 diabetes mellitus; T2DM = type 2 diabetes mellitus; TC = total cholesterol; LDL-C = low-density-lipoprotein cholesterol; BP = blood pressure; FH = familial hypercholesterolemia; eGFR = estimated glomerular filtration rate; ACR = albuminuria–creatininuria ratio; TOD = target organ damage; ASCVD = atherosclerotic cardiovascular disease.

**Table 2:** Characteristics of the study population according to CV risk stratification.

	All Participants (n = 1306)	Low–Moderate (n = 243)	High (n = 480)	Very High (n = 583)	p *
**Age (years)**	60.3 ± 12.0	50.3 ± 7.4	56.0 ± 9.3	67.9 ± 10.5	<0.001
**Sex (males)**	51.4%	23.0%	56.3%	59.2%	<0.001
**BMI (Kg/m^2^)**	28.3 ± 11.5	27.0 ± 5.0	28.3 ± 14.7	28.7 ± 10.2	0.238
**WC (cm)**	99.1 ± 11.3	92.3 ± 10.8	98.6 ± 10.7	101.5 ± 11.1	<0.001
**Obesity (BMI ≥30 Kg/m^2^)**	32.2%	26.5%	33.2%	33.3%	0.197
**DM**	12.6%	2.9%	3.1%	24.8%	<0.001
**Hypertension**	65.5%	59.3%	69.2%	65.2%	0.029
**Smoking**	37.1%	2.9%	30.6%	56.6%	<0.001
**CKD (eGFR < 60 mL/min/1.73 m^2^)**	8.0%	0.5%	8.3%	25.3%	<0.001
**LLT**	34.3%	16.0%	22.3%	52.0%	<0.001
**Antihypertensives treatment**	45.4%	49.8%	50.4%	39.5%	0.001
**SBP (mmHg)**	132.7 ± 15.3	125.7 ± 12.3	132.4 ± 14.4	135.8 ± 16.1	<0.001
**DBP (mmHg)**	78.5 ± 10.6	78.4 ± 9.2	81.1 ± 10.6	76.3 ± 10.7	<0.001
**eGFR (ml/min/1.73 m^2^)**	78.5 ± 19.5	82.3 ± 17.8	81.7 ± 18.7	71.9 ± 21.6	<0.001
**TC (mg/dL)**	194.1 ± 43.2	198.6 ± 31.9	204.8 ± 39.4	183.5 ± 47.7	<0.001
**HDL-C (mg/dL)**	54.9 ± 15.2	59.9 ± 16.1	55.2 ± 15.5	52.7 ± 14.0	<0.001
**TGs (mg/dL)**	104 (78–146)	89 (65–132)	102 (79–144)	110 (84–156)	<0.001
**LDL-C (mg/dL)**	123.3 ± 34.0	119.4 ± 24.6	132.9 ± 32.4	113.9 ± 38.7	<0.001

* *p*-values refer to trends between the different CV risk categories (low–moderate, high, very high). BMI = body mass index, WC = waist circumference, DM = diabetes mellitus, CKD = chronic kidney disease, eGFR = estimated glomerular filtration rate, LLT = lipid-lowering therapy, SBP = systolic blood pressure, DBP = diastolic blood pressure, TC = total cholesterol, HDL-C = high-density lipoprotein cholesterol, TGs = triglycerides, LDL-C = low-density lipoprotein cholesterol.

**Table 3:** Agreement of CV risk stratification according to the conventional assessment and www.humtelemed.it.

		www.humtelemed.it		
	Cardiovascular Risk	Low–Moderate (n = 255)	High (n = 462)	Very High (n = 589)
Conventional Assessment	Low–Moderate (n = 243)	240 (98.8%)	2 (0.8%)	1 (0.4%)
	High (n = 480)	15 (3.1%)	455 (94.8%)	10 (2.1%)
	Very High (n = 583)	0 (0%)	5 (0.9%)	578 (99.1%)

**Table 4:** Agreement of CV risk stratification according to the conventional assessment and www.humtelemed.it in subgroups.

Subgroups	Kappa Statistics (*p*-Value)
**Age ≥ 65 years (n = 480)**	Kappa = 0.958 (*p* < 0.001)
**Age < 65 years (n = 826)**	Kappa = 0.951 (*p* < 0.001)
**Males (n = 671)**	Kappa = 0.963 (*p* < 0.001)
**Females (n = 635)**	Kappa = 0.955 (*p* < 0.001)
**eGFR ≥ 60 mL/min/1.73 m^2^ (n = 1201)**	Kappa = 0.971 (*p* < 0.001)
**eGFR < 60 mL/min/1.73 m^2^ (n = 105)**	Kappa = 0.736 (*p* < 0.001)
**Diabetes mellitus + (n = 165)**	Kappa = 0.862 (*p* < 0.001)
**Diabetes mellitus − (n = 1141)**	Kappa = 0.962 (*p* < 0.001)
**Primary prevention (n = 1005)**	Kappa = 0.952 (*p* < 0.001)
**Secondary prevention (n = 301)**	Kappa = 0.985 (*p* < 0.001)

eGFR: estimated glomerular filtration rate.

## Data Availability

The datasets used and analyzed during the current study are available from the corresponding author upon reasonable request.

## Supplementary Materials

The following supporting information can be downloaded at https://www.mdpi.com/article/10.3390/jcm13082295/s1, Figure S1: Report of the cardiovascular risk assessment using the web app www.humtelemed.it, Table S1: Agreement of cardiovascular risk stratification according to the conventional evaluation and www.humtelemed.it in patients with eGFR < 60 mL/min (n = 105), Table S2: Agreement of cardiovascular risk stratification according to the conventional assessment and www.humtelemed.it in patients with DM (n = 165).
