# Supplementary material for: Validation of the Novel Web-Based Application HUMTELEMED for a Comprehensive Assessment of Cardiovascular Risk Based on the 2021 European Society of Cardiology Guidelines
Source: J Clin Med. 2024 Apr 16;13(8):2295. doi: 10.3390/jcm13082295 (PMC11050958; doi:10.3390/jcm13082295)
Supplement: Supplementary file 1 [file jcm-13-02295-s001.zip › jcm-2940771-supplementary.pdf]

Supplementary Figure S1. Report of the cardiovascular risk assessment using the web app www.humtelemed.it

www.humtelemed.it Name Surname DD/MM/YYYY

GLOBAL CARDIOVASCULAR RISK ASSESSMENT

**Biographical and anthropometric data**	
Age	65 years
Sex	M
Height	170 cm
Weight	75 kg
Body mass index	26.0 Kg/m²
Nationality	Italy
**Clinical and laboratory data**	
Total cholesterol	230 mg/dL
HDL cholesterol	45 mg/dL
LDL cholesterol*	161 mg/dL
Non-HDL cholesterol*	185 mg/dL
Triglycerides	120 mg/dL
Creatinine	1 mg/dL
eGFR*	78 mL/min
Systolic pressure	145 mmHg
Diastolic pressure	85 mmHg
Type 1 diabetes	No
Type 2 diabetes	Yes, diagnosed at 55 years
Genetic familial hypercholesterolemia	No
Previous cardiovascular event	No
More than two cardiovascular events in 2 consecutive years	No
**Current treatment**	
Takes medication for hypertension	No
Takes cholesterol medication	No

HUMTELEMED CLINICA MEDICA E GERIATRICA UNIVPM IRCCS-INRCA

Risk factors	
Smoking habit	Yes
Hypertension	Yes
Renal failure	No
Dyslipidemia	Yes

Outcome

Estimated current risk Very high

Based on the data you entered, your estimated probability of having a cardiovascular event in the next 10 years is greater than 10%

*marked values were estimated on the basis of the other data entered, specifically LDL cholesterol is calculated by modified Friedewald formula according to Martin-Hopkins, while non-HDL cholesterol represents all circulating atherogenic cholesterol (total cholesterol from which HDL cholesterol is subtracted). Renal filtration rate estimation was performed according to CKD-EPI formula.

Recommendations to follow after medical consultation

Non-HDL cholesterolemia collectively represents all circulating atherogenic cholesterol and is the one used to assess risk in SCORE2 charts. The values to which to bring it down are estimated to be 30 mg/dL higher than those to which LDL cholesterol should be brought down; this is especially true for those who also have triglyceridemia of 200 mg/dL and above.

Maintain blood pressure at values below 130/80 mmHg in most cases.

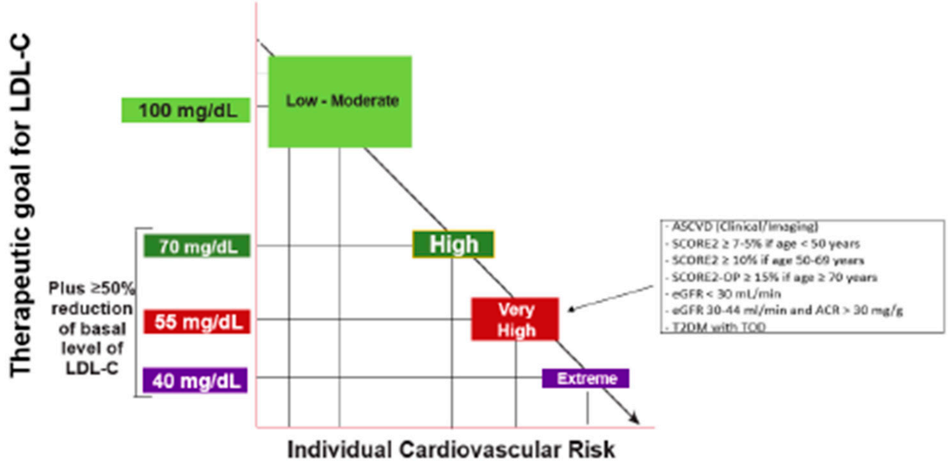

Explication of the rationale for the result on risk assessment. For primarily professional use (Physician)

The risk was estimated based on the following guidelines: ESC Guidelines on cardiovascular disease prevention in clinical practice. Eur Heart J. 42, 3227-3337, 2021; SCORE2 risk prediction algorithms: new models to estimate 10-year risk of cardiovascular disease in Europe. Eur Heart J. 42, 2439-2454, 2021; SCORE2-OP risk prediction algorithms, Eur Heart J. 42, 2455-2467; ESC/EAS Guidelines for the management of dyslipidaemias Eur Heart J 41, 111-188, 2020; ESC/ESH Guidelines for the management of arterial hypertension Eur Heart J 39, 3021-3104, 2018.

Supplemental Table S1. Agreement of cardiovascular risk stratification according to the conventional assessment and www.humtelemed.it in patients with eGFR < 60 ml/min (n. 105)

		www.humtelemed.it		
	**Cardiovascular risk**	**Low-moderate (n.12)**	**High (n.20)**	**Very-High (n.73)**
Conventional Assessment	**Low-moderate (n.1)**	1 (100.0%)	0 (0.0%)	0 (0.0%)
	**High (n.33)**	11 (33.3%)	20 (60.6%)	2 (6.1%)
	**Very-high (n.71)**	0 (0.0%)	0 (0.0%)	71 (100.0%)

Supplemental Table S2. Agreement of cardiovascular risk stratification according to the conventional assessment and www.humtelemed.it in patients with diabetes mellitus (n. 165)

		www.humtelemed.it		
	**Cardiovascular risk**	**Low-moderate (n.8)**	**High (n.10)**	**Very-High (n.147)**
Conventional Assessment	**Low-moderate (n.7)**	7 (100.0%)	0 (0.0%)	0 (0.0%)
	**High (n.15)**	1 (6.7%)	10 (66.7%)	4 (26.7%)
	**Very-high (n.143)**	0 (0.0%)	0 (0.0%)	143 (100.0%)
